# Heterologous expression of the antimyotoxic protein DM64 in *Pichia pastoris*

**DOI:** 10.1371/journal.pntd.0005829

**Published:** 2017-07-31

**Authors:** Saulo Martins Vieira, Surza Lucia Gonçalves da Rocha, Ana Gisele da Costa Neves-Ferreira, Rodrigo Volcan Almeida, Jonas Perales

**Affiliations:** 1 Laboratory of Toxinology, Oswaldo Cruz Institute, Fiocruz, Rio de Janeiro, RJ, Brazil; 2 National Institute of Science and Technology on Toxins (INCTTOX), CNPq, Brasília, DF, Brazil; 3 Laboratory of Molecular Microbiology and Proteins, Department of Biochemistry, Chemistry Institute, Federal University of Rio de Janeiro, Rio de Janeiro, RJ, Brazil; Universidad de Costa Rica, COSTA RICA

## Abstract

Snakebite envenomation is a neglected condition that constitutes a public health problem in tropical and subtropical countries, including Brazil. Interestingly, some animals are resistant to snake envenomation due to the presence of inhibitory glycoproteins in their serum that target toxic venom components. DM64 is an acidic glycoprotein isolated from *Didelphis aurita* (opossum) serum that has been characterized as an inhibitor of the myotoxicity induced by bothropic toxins bearing phospholipase A_2_ (PLA_2_) structures. This antitoxic protein can serve as an excellent starting template for the design of novel therapeutics against snakebite envenomation, particularly venom-induced local tissue damage. Therefore, the aim of this work was to produce a recombinant DM64 (rDM64) in the methylotrophic yeast *Pichia pastoris* and to compare its biological properties with those of native DM64. Yeast fermentation in the presence of Pefabloc, a serine protease inhibitor, stimulated cell growth (~1.5-fold), increased the rDM64 production yield approximately 10-fold and significantly reduced the susceptibility of rDM64 to proteolytic degradation. *P*. *pastoris* fermentation products were identified by mass spectrometry and Western blotting. The heterologous protein was efficiently purified from the culture medium by affinity chromatography (with immobilized PLA_2_ myotoxin) and/or an ion exchange column. Although both native and recombinant DM64 exhibit different glycosylation patterns, they show very similar electrophoretic mobilities after PNGase F treatment. rDM64 formed a noncovalent complex with myotoxin II (Lys49-PLA_2_) from *Bothrops asper* and displayed biological activity that was similar to that of native DM64, inhibiting the cytotoxicity of myotoxin II by 92% at a 1:1 molar ratio.

## Author summary

Snakebite envenomation causes medical emergencies that, depending on the species responsible for the bite, involve different organs and tissues. Envenomation by snakebite is a worldwide problem, and Brazil presents a high incidence of *Bothrops* bites. *Bothrops* venoms cause pathological alterations with prominent local effects, such as edema, blistering, hemorrhage, dermonecrosis and myonecrosis, usually followed by poor tissue regeneration and permanent sequelae. Bleeding, coagulopathy, cardiovascular shock and renal failure are typical systemic effects of these venoms. The clinical treatment for snakebite envenoming is intravenous administration of the specific antivenom. However, serotherapy does not efficiently protect against local tissue damage. Additional challenges faced by classical antivenom therapy include the wide antigenic variation of venoms across species and even within the same snake species and the frequent occurrence of adverse reactions that are associated with the administration of immunobiologicals. The development of new effective toxin inhibitors based on the structure of natural antiophidic proteins is an attractive therapeutic alternative. DM64 is a myotoxin inhibitor that was isolated from opossum serum, and its expression as a recombinant protein is paramount to the characterization of its structure-function relationship, an essential step toward the development of alternative strategies to better manage bothropic snakebite envenomations.

## Introduction

Accidents involving venomous snakes are medical emergencies that are often neglected in many tropical and subtropical countries [1]. Several epidemiological studies have tried to estimate the true burden of snakebite envenoming in the world. Overall, they have reported up to 5 million snake bites/envenoming per year, including tens of thousands of deaths and a much larger number of victims that are left with permanent sequelae [2–6]. The number of cases of snakebite envenomation is highest in rural regions and in cities that border on forests. Brazil also has a high level of snakebite accidents, most of which involve four predominant genera; *Bothrops* is the genus that is held accountable for the highest number of accidents [7]. According to the Brazilian Ministry of Health’s Notifiable Disease Information System (SINAN), 53,068 (provisional) *Bothrops* snakebite accidents occurred between 2013 and 2015 in the country [8].

*Bothrops* venoms contain complex mixtures of toxins that can cause the degradation of vascular basement membrane components and myonecrosis, resulting in local bleeding and tissue damage. In severe cases of envenoming, systemic bleeding, shock, hypotension and/or kidney injury may be observed, leading to high morbidity and mortality [9–11]. For longer than one century, snake envenomation has been treated using antivenoms that are based on horse antibodies. However, this remedy does not prevent local damage caused by some venomous snakes, and the antibodies can induce early or late adverse reactions [12].

The application of biochemical methods to the study of venoms has associated their pathological activities with proteins and peptides. Therefore, the study of venom proteomes (*i*.*e*., venomes) is regarded as one of the best approaches for characterizing snake venom compositions [13]. Several studies have identified and determined the relative abundances of certain classes of toxins in different snake venoms. It has also been reported that protein and peptide groups exhibit several kinds of activity on different targets, such as the cardiovascular and nervous system, blood components, and muscular and endothelial tissue [14–18]. Venomics studies of *Bothrops* have identified two of the most abundant protein groups: metalloproteases and phospholipases A_2_ (PLA_2_s). These protein groups are responsible for the most severe local clinical manifestations that are produced by these venoms, such as hemorrhage and muscular and endothelial damage [14,19–22].

Phospholipases A_2_ from snake venom have evolved into potent toxins that exhibit diverse activities such as neurotoxic, myotoxic, anticoagulant, hypotensive, cardiotoxic and edema-inducing [23]. PLA_2_s belong to one of the five principal groups that catalyze the Ca^2+^-dependent hydrolysis of the acyl ester at the sn-2 position of glycerophospholipids. Basic myotoxic phospholipases A_2_ are responsible for tissue degradation and necrosis at the bite site. These myotoxins bind to the plasma membrane of skeletal muscle cells, generating muscle necrosis. Some PLA_2_s contain a critical substitution at the calcium-binding site (Asp^49^ to Lys^49^) that renders them non-catalytic, yet they conserve their myotoxic activity [24–29].

Many studies have sought alternative sources of natural venom inhibitors to complement the action of antivenoms, particularly to help neutralize local tissue damage. Some reports have identified some natural components that inhibit PLA_2_ myotoxins from several snake venom species [30–33]. Additionally, several studies have investigated myotoxic inhibitors that have been isolated from reptile or mammalian sera [34–36]. DM64 is a myotoxin-specific inhibitor isolated from *D*. *aurita* (opossum) serum and has been characterized as an acidic glycoprotein of 64 kDa in size. DM64 has been structurally classified as a member of the immunoglobulin supergene family, showing five immunoglobulin-type domains that are similar to α1B-glycoprotein [35,37]. DM64 forms a noncovalent soluble complex and efficiently inhibits the myotoxic activity of both inactive Lys49-PLA_2_ and active Asp49-PLA_2_ but does not inhibit the catalytic activity of the latter [35].

Currently, many alternative treatments are being developed to supplement antivenom serum treatments and prevent local tissue damage. Several newly discovered natural antiophidic molecules from plant extracts have been reported [30–33]. However, their pharmacological reliability has not yet been demonstrated because these inhibitors have different functional groups that could interact with different molecular targets[30,32,33]. Myotoxin inhibitors isolated from snake and mammalian sera seem to be more specific, making their recombinant expression a promising strategy for new therapeutic developments [35–38].

DM64 is an efficient myotoxin inhibitor that was isolated from *D*. *aurita* serum, and the heterologous expression of this glycoprotein could be a breakthrough in the development of *Bothrops* envenomation treatment. Thus, the aim of this study was to express rDM64 using *Pichia pastoris*. The biological activity of the recombinant inhibitor on the myotoxin Lys49-PLA_2_ from *B*. *asper* was analyzed.

## Methods

### Reagents

Yeast extract, peptone, biotin, dextrose, agar, peroxidase-conjugated anti-rabbit secondary antibody, Pefabloc SC (4-(2-aminoethyl)-benzenesulfonyl fluoride), sorbitol, EDTA (ethylenediaminetetraacetic acid) and DAB (3,3’-diaminobenzidine) substrate kit were purchased from Sigma (Missouri, USA). Glycerol and methanol were supplied by VETEC (Rio de Janeiro, Brazil). *Pichia pastoris* X-33, pPICZαA, and Zeocin were purchased from Invitrogen (California, USA). Restriction enzymes and PNGase F were furnished by New England Biolabs (Massachusetts, USA). Electroporation cuvettes, TMB (3,3’,5,5’-tetramethylbenzidine) EIA substrate kit and low-range SDS-PAGE standards were obtained from Bio-Rad Laboratories (California, USA). Trypsin was purchased from Promega (California, USA). Pierce Glycoprotein Staining kit, DMEM (Dubelcco’s Modified Eagle Medium), and fetal bovine serum were purchased from Thermo Fisher Scientific (Massachusetts, USA).

### Gene synthesis and plasmid construction

The *DM64* gene sequence [35] was synthesized by Epoch Life Science (Missouri, USA). The gene was cloned without a signal peptide into the expression vector pPICZαA, which contained an alcohol oxidase 1 (AOX1) promoter. The gene sequence was cloned in-frame with the *S*. *cerevisiae* α-factor secretion sequence that is present in pPICZαA. A stop codon was inserted before the *c-myc* epitope and the polyhistidine (6x His) tag. The resulting plasmid (named pPICZαA-DM64) was linearized with *SacI* (a site present in the *AOX1* promoter) prior to its transformation into *P*. *pastoris* X-33 cells via electroporation.

### Yeast selection and transformant screening

Transformed cell suspensions were diluted to guarantee the growth of the well-separated colonies on the surface of a solid medium. The colonies were grown in YPDS (1% yeast extract, 2% peptone, 2% dextrose, 1 M sorbitol, and 2% agar) medium containing 0.1 mg/mL Zeocin at 30°C for 3 to 5 days. A second selection was then performed in liquid YPD medium containing 0.1 mg/mL Zeocin at 30°C, 250 rpm for 24 hours. Fourteen clones that grew under these last conditions were submitted to a second screening by using increasing concentrations of antibiotics (0.2, 0.4, 0.6, 0.8, and 1 mg/mL Zeocin). The cells were grown at 30°C and 900 rpm, for 48 hours.

### Protein expression in shake-flask culture

*Pichia pastoris* with the multicopy expression vector pPICZαA-DM64 was initially grown in BMGY medium, which contains glycerol as a carbon source (1% yeast extract, 2% peptone, 1.34% YNB, 4 x 10^−5^% biotin, 100 mM potassium phosphate, pH 6, and 1% glycerol) at 30°C and 250 rpm. The culture grew until 50-fold diluted aliquots reached an OD_600nm_ of 0.36, which may have taken up to 24 hours. The culture medium was then changed to BMMY, which contains methanol for the purposes of induction and to be used as a carbon source (1% yeast extract, 2% peptone, 1.34% YNB, 4 x 10^−5^% biotin, 100 mM potassium phosphate, pH 6, and 1% methanol). The culture flask was then incubated at 30°C and 250 rpm, for 144 and 264 hours. One percent methanol was added every 24 hours to maintain protein expression. Yeast culture with 0.2 mM Pefabloc was added to the BMMY medium every 24 hours. The culture was centrifuged at 5,000xg for 20 minutes at 4°C, and the supernatant was collected and stored at -20°C. Aliquots of the culture were analyzed by using 12% SDS-PAGE gels [39] that were stained with silver nitrate [40]. The following low-range SDS-PAGE standards were used: phosphorylase b (97.4 kDa), serum albumin (66.2 kDa), ovalbumin (45 kDa), carbonic anhydrase (31 kDa), trypsin inhibitor (21.5 kDa), and lysozyme (14.4 kDa). Molecular mass estimates were calculated using Image Master 2D Elite software (GE Healthcare, version 4.01).

### Quantification of rDM64 in expression medium by immunoassay

The expression medium (50 μL) was alkalized by adding 1 M sodium carbonate buffer (10 μL), and each aliquot that was taken at a different time after induction (0–264 hours) was incubated at 37°C for 2 hours in a 96-well polystyrene plate. The wells were washed three times with wash buffer (PBS buffer and 0.1% (v/v) Tween 20). Then, the wells were blocked with PBS buffer containing 5% (w/v) non-fat dry milk and incubated at 37°C for 2 hours. The wells were washed three times with wash buffer containing 5% (w/v) non-fat dry milk and then incubated for 1 hour at 37°C with fresh wash buffer containing 5% (w/v) non-fat dry milk and 100 μL of polyclonal anti-DM64 antibodies (0.23 mg/mL), prepared as previously described [41], except for the use of DM64 as antigen, instead of anti-hemorrhagic proteins. After this incubation, the wells were washed three times with wash buffer and then incubated at 37°C for 1 hour with 100 μL of peroxidase-conjugated secondary anti-rabbit antibodies (1:30,000) in wash buffer that contained 5% (w/v) non-fat dry milk. Finally, the wells were washed three times with wash buffer and then incubated for 20 minutes at room temperature with 100 μL of the Single Component TMB (3,3’,5,5’-tetramethylbenzidine) EIA Substrate kit. The reaction was stopped by the addition of 1 N H_2_SO_4_, and the absorbance at 450 nm was recorded. Native DM64 (0.1, 0.2, 0.4, 0.8 and 1.6 μg/50 μL) was used to build the standard curve.

### Protein trypsinization and identification by mass spectrometry

Bands in silver-stained SDS-PAGE gels were digested as previously described [42], with modifications. They were first incubated twice with 100% (v/v) acetonitrile for 15 minutes and then dried under vacuum for 15 minutes. The bands were reduced by incubating the samples with 65 mM 1,4-dithiothreitol at 56°C for 30 minutes. The reduction buffer was removed, and the bands were washed twice in 100 mM ammonium bicarbonate for 10 minutes, followed by a 5 minute wash in 100% acetonitrile. The bands were dried under vacuum for 15 minutes; 20 ng/μL trypsin was added, and they were then incubated for 45 minutes on ice. Excess trypsin was removed, and 40 mM ammonium bicarbonate was added. Hydrolysis proceeded overnight at 37°C and then for an additional 45 minutes at 56°C. Finally, the digested products were desalted using tip columns that were packed with Poros R2 resin (Applied Biosystems) and equilibrated with 0.1% (v/v) formic acid in water. After washing away nonbound material (10 x 20 μL) by using equilibrium buffer, the peptides were eluted using 0.1% (v/v) formic acid in 50% (v/v) acetonitrile and dried under vacuum. Desalted tryptic peptides were redissolved in 1% (v/v) formic acid, and 4 μL of each sample was loaded onto a home-made capillary guard column (2 cm x 100 μm i.d.) that was packed with 5 μm, 200 Å Magic C18 AQ matrix (Michrom Bioresources, Auburn, CA, USA). Peptide fractionation was performed on an analytical column (10 cm x 75 μm i.d.) with a laser pulled tip (~5 μm) that was packed with the same matrix and coupled to an Ultimate 3000 RSLCnano chromatography system (Thermo Fisher Scientific, Waltham, MA, USA). The analysis was conducted using an LTQ Orbitrap XL mass spectrometer (Thermo Fisher Scientific, Waltham, MA, USA) with a capillary temperature of 200°C, tube lens voltage of 100 V and a nanoESI source with a spray voltage set to 1.9 kV and no sheath gas. Samples were added with water containing 0.1% (v/v) formic acid into the trap column at 2 μL/min, while the chromatographic separation occurred at 0.2 μL/min. The peptides were eluted with a 2–40% (v/v) acetonitrile gradient in 0.1% (v/v) formic acid over 32 minutes, which then ramped to 80% acetonitrile in 4 minutes and was followed by a final washing step at 80% acetonitrile for an additional 2 minutes. The mass spectrometer operated in data-dependent mode, using the following settings: for MS1, a 300–1700 *m/z* scan range, 1 x 10^6^ automatic gain control (AGC), 500 ms maximum injection time (IT), centroid mode acquisition and resolution of 60,000 (FWHM at *m/z* 400). Up to 7 of the most intense precursor ions in each survey scan were selected for CID fragmentation in the LTQ using 35% normalized collision energy (NCE). MS2 analysis was performed with the following parameters: 1 x 10^4^ AGC, 100 ms IT, and centroid mode acquisition. The isolation window was 2 *m/z*, and only precursor ions with a charge state ≥ 2 were selected for fragmentation, setting the dynamic exclusion to 45 s. The spectrometer was calibrated using a calibration mixture composed of caffeine, peptide MRFA, and Ultramark 1621, as recommended by the instrument manufacturer.

### Mass spectrometry data analysis

The *Pichia pastoris* sequence database was obtained from UniProt (Proteome ID UP000000314, containing 5,073 protein sequences). The sequences of DM64 (UniProt Q8MIS3, excluding the signal peptide), DM43 (UniProt P82957) and common contaminants (ftp://ftp.thegpm.org/fasta/cRAP) were also included in the protein database. Peaks Studio software (version 7.5) was used for *de novo* sequencing assisted database search [43] using the following parameters: monoisotopic masses, carbamidomethylation of cysteine (fixed modification), oxidation of methionine (variable modification), semi-tryptic digestion, 10 ppm peptide mass error tolerance, 0.6 Da fragment mass tolerance, up to 2 variable modifications per peptide and a maximum of 2 missed cleavages. The data were filtered using the PEAKS decoy fusion approach, and false discovery rates (FDR) were set to a maximum of 1% at the peptide level. Only proteins that were identified with at least 2 unique peptides were accepted (FDR values at the protein level ≤1%).

### Identification of rDM64 by Western blotting

Expression products in the supernatant and purified rDM64 were separated by 12% SDS-PAGE gel [39], and the proteins were transferred to a 0.45 μm nitrocellulose membrane (GE Healthcare Life Science, USA) using ice-cold transfer buffer (25 mM Tris-HCl, pH 8, 192 mM glycine, and 20% methanol). The membrane was blocked in TBS-Tween (25 mM Tris-HCl, pH 8.0, 140 mM NaCl, 2 mM KCl, and 0.05% Tween 20) containing 5% (w/v) non-fat dry milk overnight at 4°C. The membrane was then washed three times in TBS-Tween and was incubated for 2 hours at room temperature with diluted [1:100 (v/v)] crude rabbit serum that was raised against native DM64 [41]. The membrane was subsequently washed three times in TBS-Tween and was incubated with 1:5000 (v/v) peroxidase-conjugated secondary anti-rabbit antibody. The rDM64 bands were visualized using a DAB substrate kit. The following prestained low-range SDS-PAGE standards were used: phosphorylase B (103 kDa), bovine serum albumin (77 kDa), ovalbumin (50 kDa), carbonic anhydrase (34 kDa), and soybean trypsin inhibitor (28.8 kDa).

### Purification of rDM64

The expression medium that contained the recombinant protein was concentrated using Amicon Ultra 15 mL centrifugal filters (Merck Millipore, USA) with a cutoff of 10 kDa. The expression medium was exchanged to buffer (20 mM Tris-HCl pH 7.5), and the sample was injected into a column that had already been equilibrated with the same buffer. This column, a HiTrap NHS (7 x 25 mm, GE Healthcare Life Sciences, USA) activated column that contained myotoxin II (from *B*. *asper*) that had been immobilized according the manufacturer’s instructions, was connected to an ÄKTA Purifier chromatography system (GE Healthcare Life Sciences, USA). The rDM64 fraction was eluted at a flow rate of 1 mL/min with 0.1 M glycine, pH 2.7, and collected over 1 M Tris to neutralize the solution. The fraction was concentrated using Amicon Ultra 4 mL centrifugal filters (Merck Millipore, USA) with a cutoff of 10 kDa. The recombinant protein fraction buffer was exchanged to the equilibration buffer of a Mono Q GL column (5 x 50 mm, GE Healthcare Life Sciences, USA) (20 mM Tris-HCl, pH 7.5). The recombinant protein was eluted during a 20 minute 0–1 M NaCl linear gradient at a flow rate of 0.5 mL/min. Protein concentrations in the collected fractions were determined using the bicinchoninic acid assay (BCA method) with BSA as standard [44]. Their electrophoretic profiles were analyzed by using SDS-PAGE under reducing conditions [39].

### Removal of carbohydrate residues from rDM64

PNGase F was used to cleave between the innermost N-acetylglucosamine (GlcNAc) residues and the asparagine residues to which they are linked in this high-mannose-type recombinant glycoprotein. Initially, 3 μg of purified rDM64 was mixed with 0.5% SDS and 40 mM DTT, and the solution was incubated at 100°C for 10 minutes. Then, 50 mM sodium phosphate, pH 7.5, 1% NP-40, and 1,000 U of PNGase F were added, and the solution was incubated at 37°C for 1 hour. The reaction was stopped by adding 5 μL of denaturing buffer (5X, final concentrations: 0.3 M Tris-HCl, pH 6.8, 2% SDS, and 0.1 M DTT), and the separation of the reaction products was visualized using 12% SDS-PAGE [39] gel with silver staining [40]. Native DM64 was used as a positive control in this experiment.

### Staining of rDM64 glycans in SDS-polyacrylamide gels

The presence of sugars in rDM64 was determined with the periodic acid-Schiff method using the Pierce Glycoprotein Staining kit. The staining of 5 μg of rDM64 was done following the manufacturer’s instructions. Native DM64 was used as a positive control in this experiment. All samples were analyzed using 12% SDS-PAGE gels [39].

### Complex formation

The interaction between myotoxin II (PLA_2_-Lys49) from *B*. *asper* and rDM64 was monitored by using a 12% native PAGE gel [39]. The myotoxin-rDM64 complex (2:1 molar ratio) was incubated in 20 mM Tris-HCl, pH 7.5, and 150 mM NaCl at 37°C for 30 minutes. Native DM64 was used as a positive control in this experiment and the gel was stained with silver nitrate [40].

### Cytotoxicity assay

Murine myoblast cell line C2C12 (ATCC CRL-1772), which can fuse and differentiate into myotubes, was used. The cells were grown in Dulbecco’s Modified Eagle Medium (DMEM) that was supplemented with 44 mM sodium bicarbonate, 19.5 mM glucose, 2 μM L-glutamine, 100 U/mL penicillin, 0.1 mg/mL streptomycin, and 10% fetal bovine serum (FBS) in a humidified atmosphere with 5% CO_2_ at 37°C. Cells were harvested from near-confluent cell monolayers grown in 25 cm^2^ bottles. After detaching the cells by using 1500 U/mL trypsin containing 5.3 mM EDTA for 2 minutes at 37°C, the resuspended cells were seeded in 96 wells at an approximate initial density of 1 x 10^4^ cells/well in the same medium. Upon reaching near confluence in 3 days, the growth medium was replaced with a differentiation medium (DMEM supplemented with 1% FBS). When multinucleated myotube cells were observed (after 6 days of culture), they were utilized in a cytotoxicity assay [25]. Myotoxin II and recombinant or native DM64 were preincubated (1:1, 2:1 and 4:1, mol:mol) for 30 minutes at 37°C in 150 μL of DMEM supplemented with 1% FBS. After aspirating the old medium, the samples were added to the cell cultures that were growing in 96 wells to yield a total volume of 150 μL/well. After three hours of incubation at 37°C with 5% CO_2_, 20 μL aliquots of supernatant were collected to determine lactate dehydrogenase activity. Controls for 0% and 100% toxicity consisted of assay medium and 0.1% Triton X-100 in assay medium, respectively. The results are presented as the mean ± standard deviation (n = 3). Statistical analyses were by one-way ANOVA followed by Student–Newman–Keuls post hoc test (GraphPad Prism 5.0 software). P-values of 0.05 or less were considered significant.

## Results

**Fig 1:**
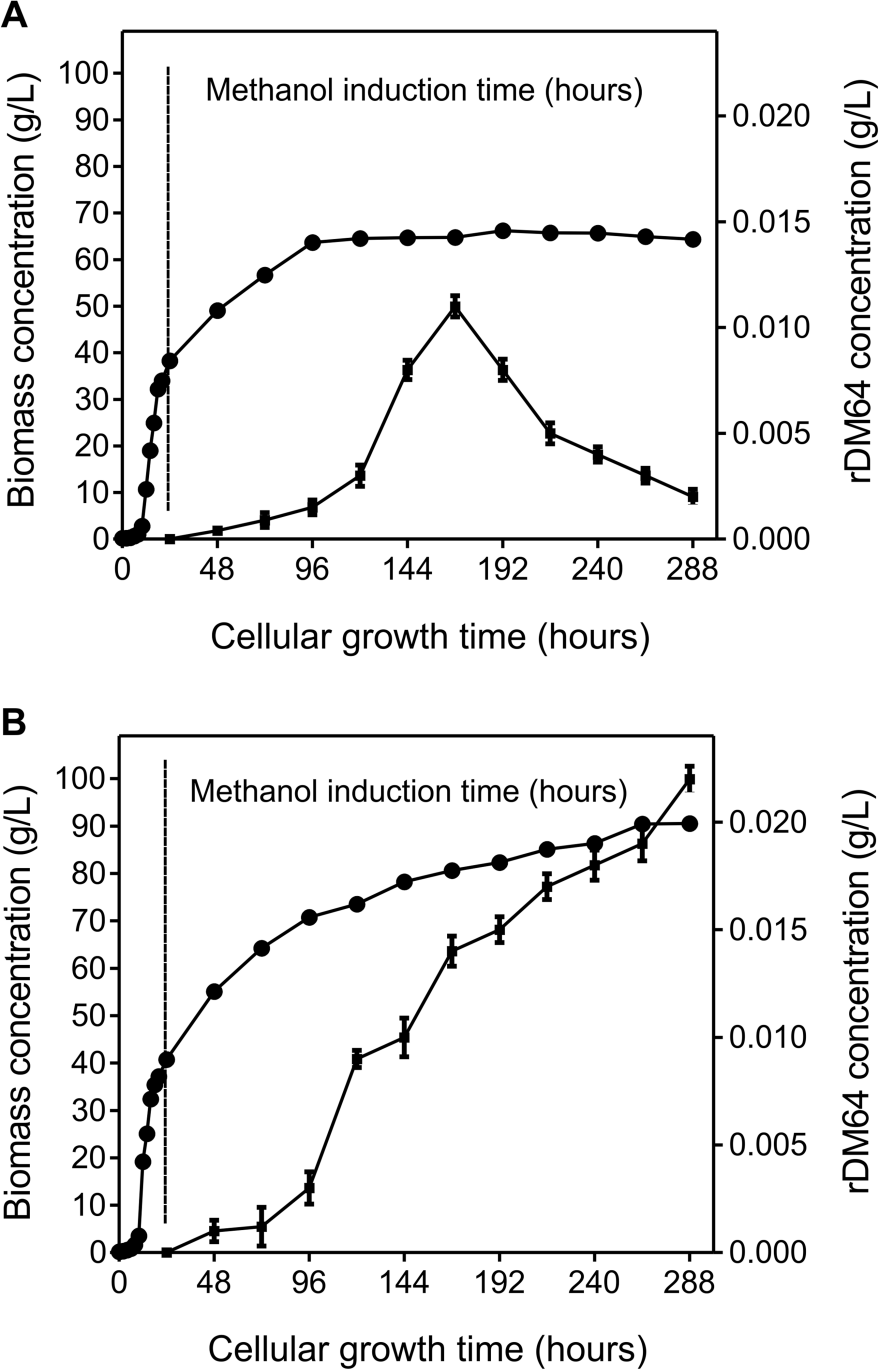
Time-course profiles of rDM64 secretion in *Pichia pastoris* culture during fermentation. *P*. *pastoris* was grown at 30°C and 250 rpm for 288 hours. 0–24 hours: glycerol batch phase. 24–288 hours: methanol induction (fed-batch phase); 1% methanol was added every 24 hours, for 264 hours. Dry cell weight/biomass (circle) and rDM64 concentration (square) were determined in the absence **(A)** or presence **(B)** of 0.2 mM Pefabloc. The concentration of rDM64 was measured by immunoassay in the culture medium and was performed in triplicate. Error bars represent the standard deviation of the mean.

Recombinant glycoprotein production was carried out in a 1 L culture flask using a two-step growth protocol that consisted of a glycerol batch phase and a methanol fed-batch phase. During the glycerol batch phase, *P*. *pastoris* was cultivated at 30°C for 24 hours, and the cellular concentration reached approximately 40 g/L. The methanol fed-batch phase was maintained at 30°C, and the induction lasted 264 hours, yielding 288 hours of total cell growth time. *Pichia pastoris* cells were grown in methanol in the absence (Fig 1A) or presence (Fig 1B) of 0.2 mM Pefabloc, a serine protease inhibitor. During the methanol fed-batch phase, cell growth without Pefabloc was maximum at 72 hours (96 hours of total cell growth time). From 72 to 264 hours of methanol induction, the cell concentration was constant, around 63 g/L (Fig 1A). On the other hand, in the presence of Pefabloc, the cell growth continued after 72 hours, reaching a biomass yield of 90 g/L at 264 hours (288 hours of total cell growth time)(Fig 1B).

**Fig 2:**
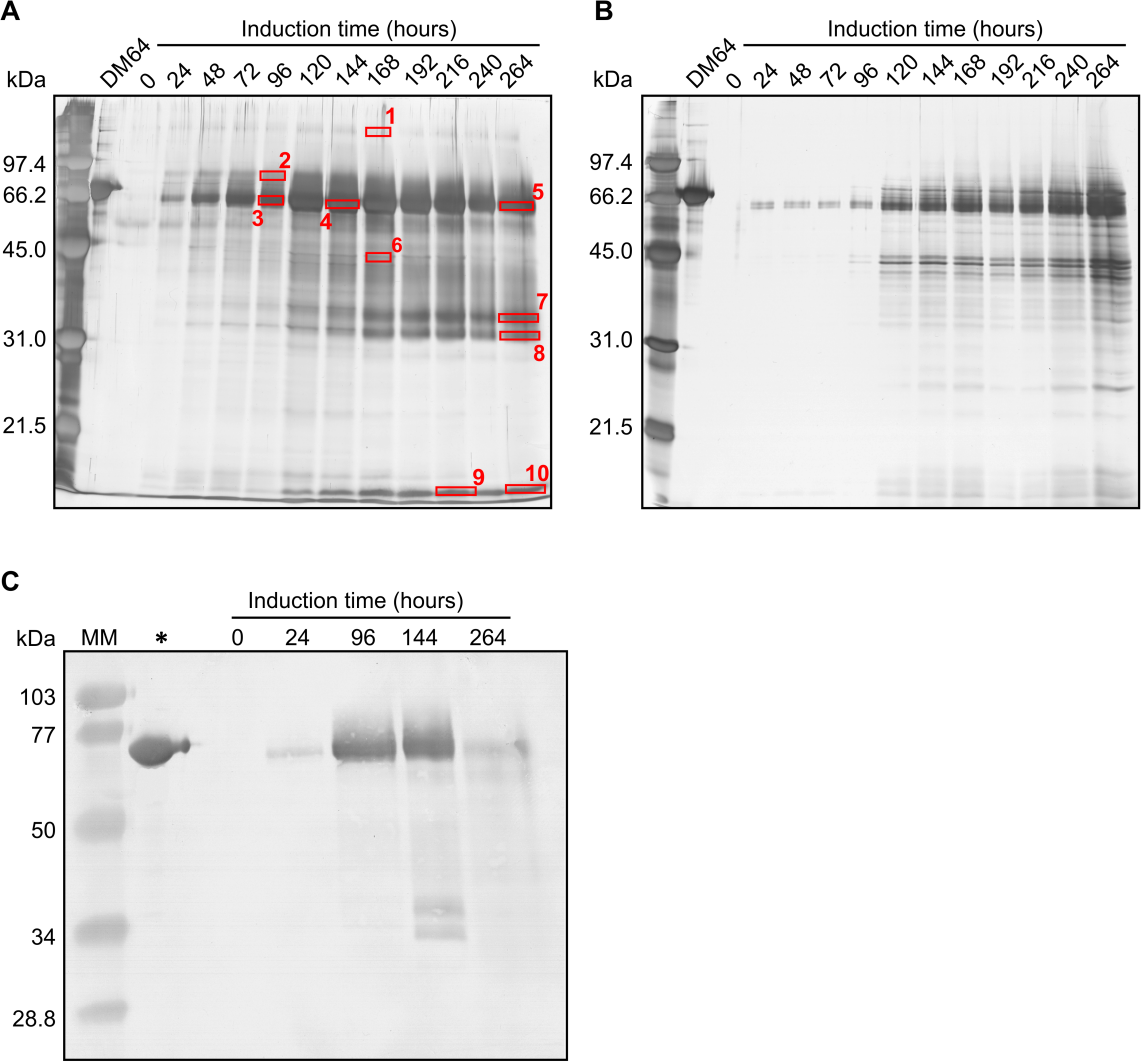
**SDS‑PAGE (12%) analysis of culture supernatant from *P*. *pastoris* fermentation during the growth time in the absence (A) or presence (B) of 0.2 mM Pefabloc.** Aliquots of *P*. *pastoris* culture media (~ 14 μL) that were taken at different time points were loaded into each lane under reducing conditions. The gels were silver stained, and the proteins in the gel bands that were indicated by red rectangles were identified by MS/MS (S1 Table). (C) Identification of rDM64 in yeast culture medium by immunoblotting using polyclonal antibodies raised against native DM64. Different induction times were assayed in the absence of Pefabloc. The asterisk indicates native DM64 purified from opossum serum, which was used as the positive control. MM represents prestained molecular mass markers.

The use of a serine protease inhibitor during fermentation was important to not only positively stimulate *P*. *pastoris* growth but also improve rDM64 expression yields. After 264 hours of methanol induction, the rDM64 concentration in the fermentation medium increased from 0.002 g/L without Pefabloc (Fig 1A) to approximately 0.02 g/L in the presence of Pefabloc (Fig 1B). Without protease inhibitor, the recombinant protein concentration in the fermentation medium increased until 144 hours of induction (168 hours of cell growth time) were reached and decreased thereafter until 264 hours (288 hours of cell growth time)(Fig 1A). The 65 kDa band in the SDS-PAGE gel that corresponds to rDM64 shows the same expression kinetics (Fig 2A), as does immunoblotting (Fig 2C). Several bands with lower molecular masses whose intensities progressively increase during 264 hours of methanol induction can also be observed by SDS-PAGE (Fig 2A). On the other hand, the amount of rDM64 in the culture medium containing Pefabloc favored a progressive increase (Fig 1B), which corresponds to the behavior of the main 65 kDa band observed by SDS-PAGE during the same time interval; there were also smaller amounts of protein bands with lower relative molecular masses (Fig 2B).

The bands indicated in red in Fig 2A were digested in gel, and the resulting peptides were analyzed by mass spectrometry (S1 Table). Database searches using the masses of these peptides and their fragment ions led to the unequivocal identification of the 65 kDa bands (bands # 3–5) as full-length DM64. The identity of DM64 was further confirmed by Western blotting using polyclonal antibodies, as shown in Fig 2C. Additional fainter bands with higher (bands # 1–2) and lower (bands # 6–10) relative molecular masses were also identified as DM64. In this last case, partial proteolytic degradation of rDM64 seems the most likely explanation, given the reduced abundance of these bands in the presence of Pefabloc. Higher molecular mass bands (band #1: 136 kDa and band #2: 88 kDa, Fig 2A) were also identified as DM64, and two explanations may be envisaged: either there is a strongly aggregated form of rDM64 and/or the recombinant protein has been expressed with different levels of glycosylation.

**Fig 3:**
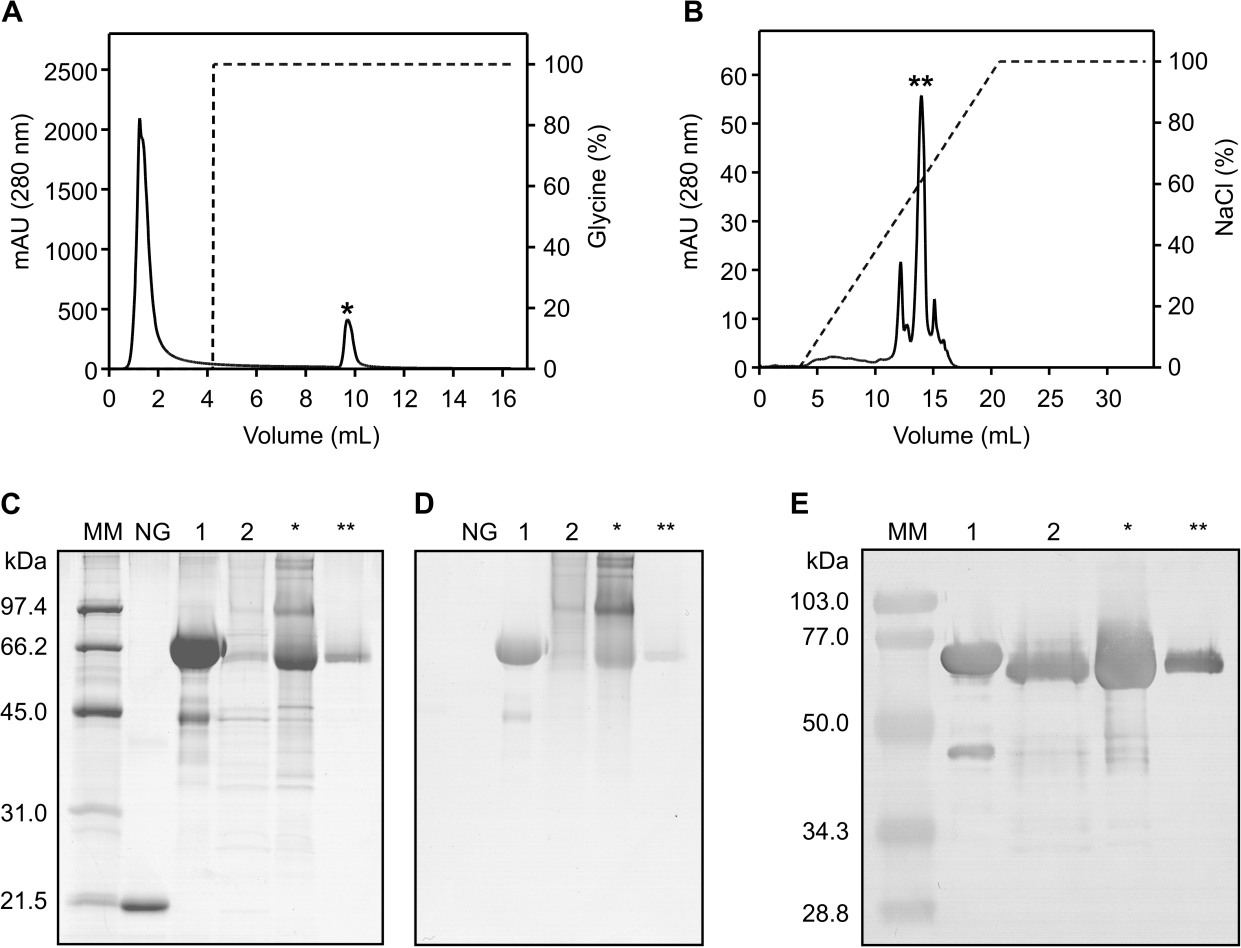
Characterization of rDM64 protein purified from yeast medium. (A) Purification of rDM64 from the culture medium supernatant by affinity chromatography with immobilized myotoxin II. (B) After elution, the bound fraction (indicated by an asterisk) was further purified by anion-exchange chromatography using a Mono Q column. The chromatographic fractions were analyzed by 12% SDS-PAGE under reducing conditions and were stained with Coomassie blue (C) and periodic acid-Schiff reagent (D) or by immunoblotting with polyclonal antibodies raised against DM64 (E). Lane 1, native DM64 (5 μg). A typical degradation product ~ 45 kDa in size, generated by sample manipulation, can also be observed; lane 2, crude culture medium. Asterisks indicate the chromatographic fractions and are shown in panels A and B (5 μg/lane). NG, non-glycosylated protein (soybean trypsin inhibitor) used as negative control for the periodic acid-Schiff staining. MM, molecular mass markers.

The culture medium containing the recombinant inhibitor was fractionated by liquid chromatography using an affinity column conjugated with myotoxin II, a Lys49-PLA_2_ isolated from *B*. *asper* venom [45]. DM64 specifically binds to the immobilized myotoxin, and for this reason, this first purification step efficiently removed most impurities (culture medium proteins), allowing for the recovery of a protein fraction that was enriched in rDM64 (Fig 3A). However, the SDS-PAGE profile of the sample under reducing conditions showed that the chromatographic peak corresponding to rDM64 was still heterogeneous, with both higher and lower molecular mass protein bands co-purified with full-length rDM64 (Fig 3C). Native DM64 is an acidic protein with a pI of 4.5 [35]; thus, a second chromatography step using an anion-exchange column was necessary to improve protein purification, as shown in Fig 3 (panels B and C). rDM64 was noted to be glycosylated following positive staining with periodic acid-Schiff reagent (Fig 3D) and was recognized by polyclonal antibodies raised against native DM64 (Fig 3E).

**Fig 4:**
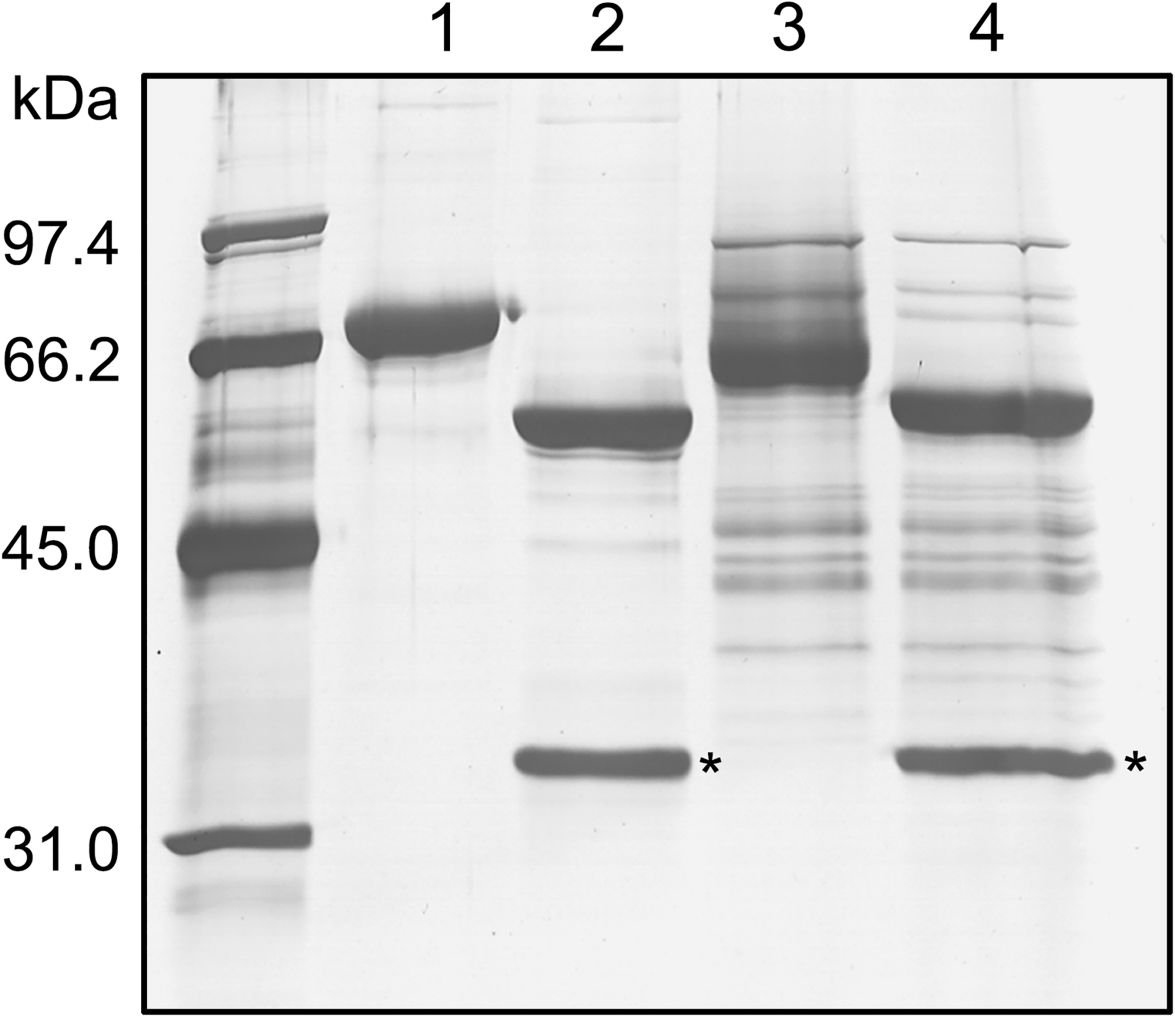
Enzymatic deglycosylation of DM64 by PNGase F analyzed by SDS-PAGE. Native DM64 and affinity-purified rDM64 proteins were denatured before the removal of the carbohydrate residues. The glycoproteins were incubated with 1,000 U of recombinant PNGase F at 37°C for 1 hour. Lane 1, native DM64 before deglycosylation; lane 2, native DM64 after deglycosylation; lane 3, rDM64, purified by affinity column, before deglycosylation; lane 4, rDM64, purified by affinity column, after deglycosylation. The asterisks indicate protein bands corresponding to the PNGase (36 kDa). Samples were analyzed under reducing conditions on 12% gels that were stained with silver nitrate.

rDM64 was treated with the glycosidase PNGase F to remove N-linked oligosaccharides (Fig 4). Due to the limited amount of homogeneous protein, a partially purified fraction that was purified by affinity column was used instead of the protein preparation that was obtained by anion-exchange chromatography. Both native DM64 (71 kDa)(Fig 4, lane 1) and rDM64 (65 kDa)(Fig 4, lane 3) proteins were submitted to carbohydrate removal. Deglycosylated rDM64 showed a main molecular band at 56 kDa (Fig 4, lane 4), which is closer to the molecular mass of native DM64 without the glycan moiety (55 kDa)(Fig 4, lane 2). The theoretical average molecular mass of DM64 based on its primary sequence is 53.3 kDa (calculated using http://web.expasy.org/compute_pi). Therefore, given the accuracy (± 10%) of molecular mass estimates by SDS-PAGE [46], the molecular masses of deglycosylated DM64 (native/recombinant) proteins are in close agreement with the expected value.

**Fig 5:**
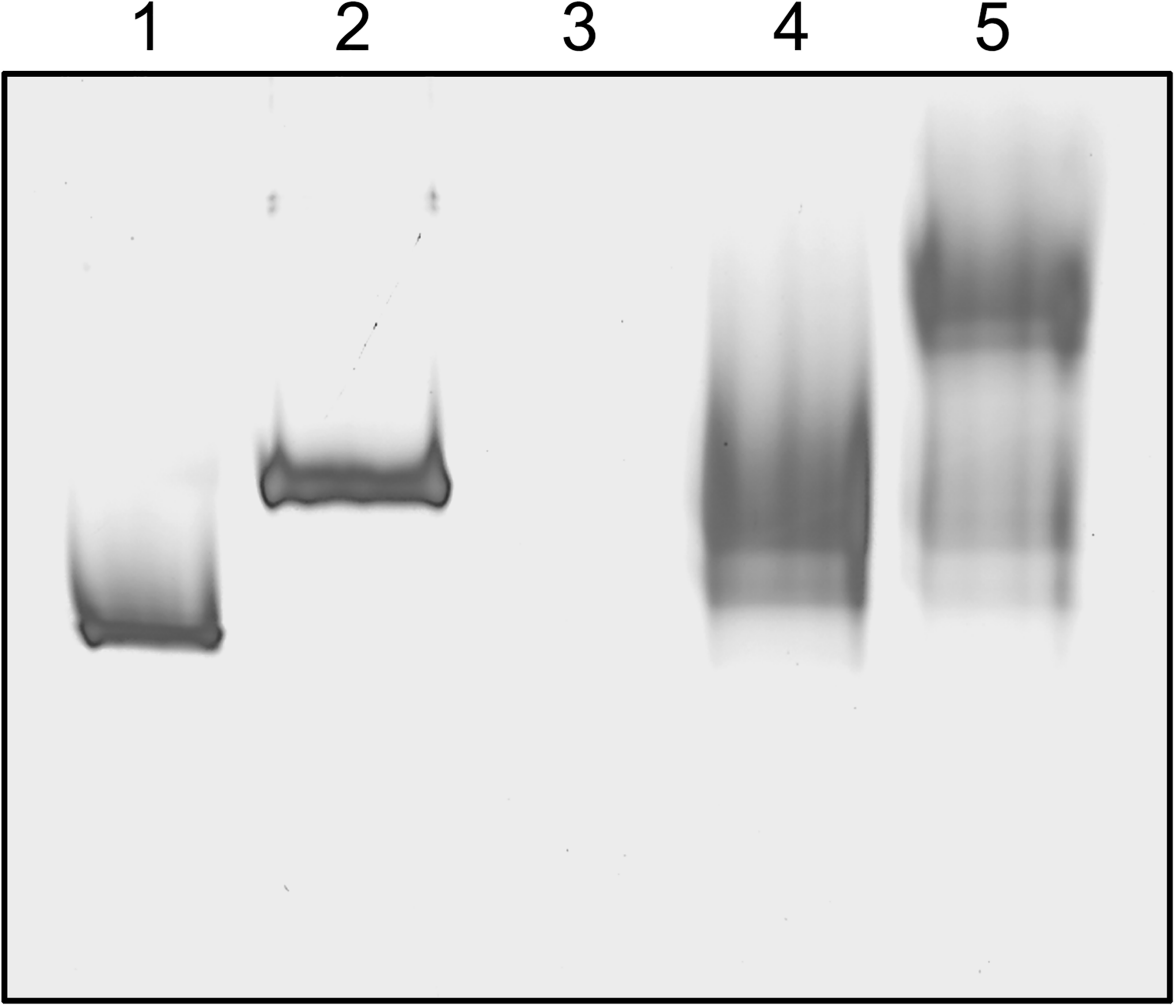
Complex formation between myotoxin II (Lys49-PLA_2_) from *B*. *asper* venom and anion-exchange purified rDM64. Myotoxin II (mt II) and rDM64 were mixed at a 2:1 (mol:mol) ratio and incubated for 30 minutes at 37°C; this sample was then analyzed using 12% native PAGE stained with silver nitrate. Lane 1, native DM64 (2.3 μg); lane 2, mt II-native DM64 complex (2:1, mol:mol); lane 3, mt II (1 μg); lane 4, rDM64 (2.3 μg); and lane 5, mt II-rDM64 (2:1, mol:mol).

The ability of the recombinant inhibitor to bind to myotoxin II (Lys49-PLA_2_) from *B*. *asper* venom was analyzed. Myotoxin II (mt II) and rDM64 were mixed at a 2:1 (mol:mol) ratio, and complex formation was monitored by electrophoresis under native conditions. Fig 5 (lane 5) shows a band with an electrophoretic mobility that corresponds to the complex. Native DM64 was used as a control for complex formation (Fig 5, lane 2). Due to the basic nature of myotoxin-II (pI 9.1)[47], it does not enter the gel under native conditions (Laemmli buffer system without SDS). The band corresponding to the rDM64-mt II complex (Fig 5, lane 5) shows a different mobility than that of DM64-mt II (Fig 5, lane 2) due to the difference in the net charges of the proteins; this factor is a major variable that influences electrophoresis mobility on native/non-denaturing gels. rDM64 has a different charge than that of native DM64 due to the absence of sialic acid, which is negatively charged.

**Fig 6:**
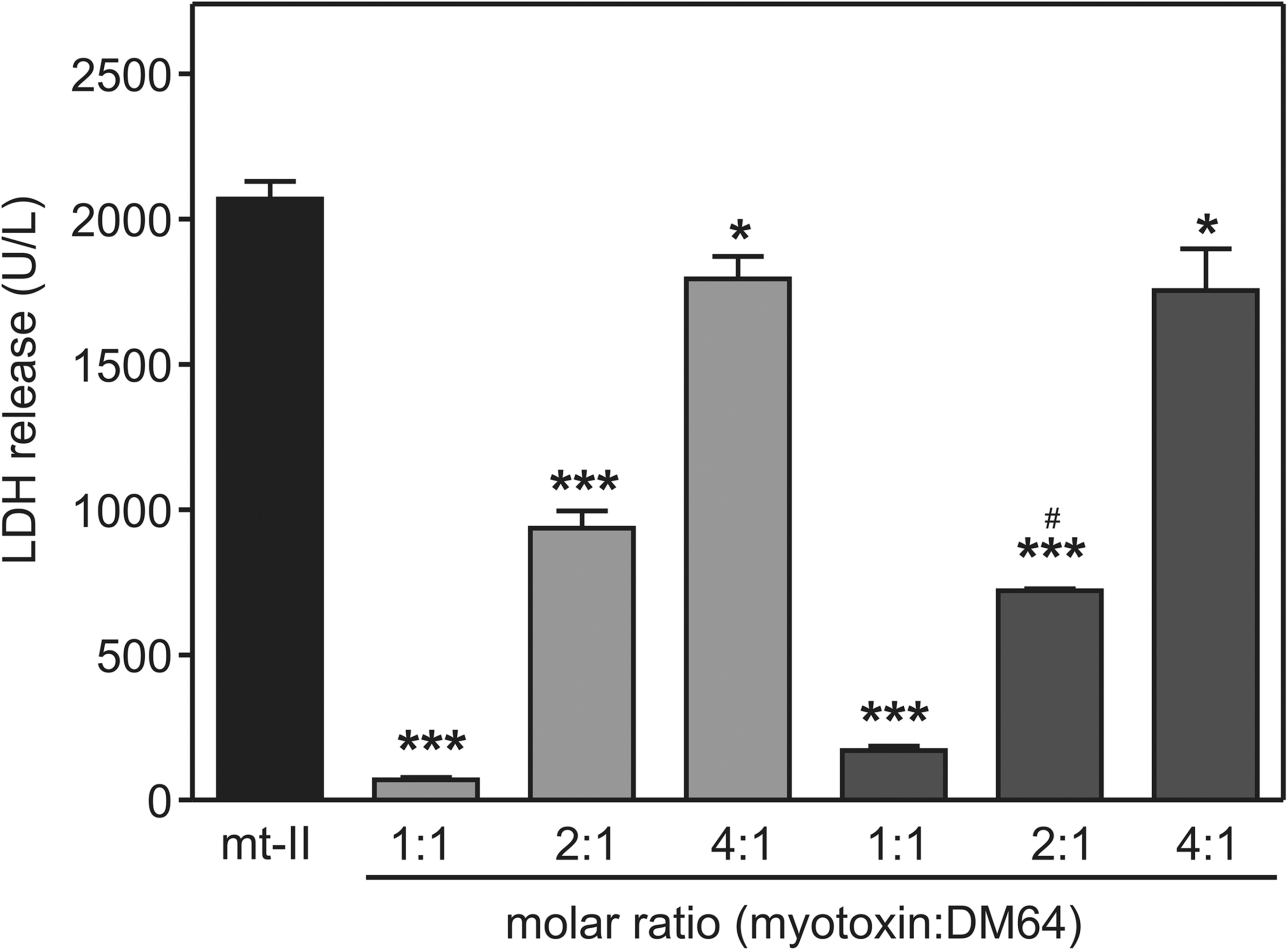
Inhibitory activity of rDM64 purified by affinity chromatography on the cytotoxic activity of *B*. *asper* myotoxin II upon C2C12 myotubes. Myotoxin II (mt II) was incubated with native DM64 or rDM64 for 30 minutes at 37°C. The mixture was then added to a C2C12 myotube culture. Cytolysis was monitored based on the amount of lactate dehydrogenase that was released into the medium 3 hours after the addition of myotoxin alone (black) or myotoxin in complex with native DM64 (light grey) or rDM64 (dark grey). The bars represent the mean ± SD of triplicate cell cultures. * 0.01 < P < 0.05 and *** P < 0.001 when compared to the myotoxin control. The inhibition effects of equivalent doses of native and recombinant DM64 were also compared (^#^ 0.01 < P < 0.05). Statistical analyses were by one-way ANOVA followed by Student–Newman–Keuls post hoc test.

The inhibition of the cytotoxicity induced by myotoxin II from *B*. *asper* on C2C12 myogenic cells by rDM64 was also evaluated. The toxin-inhibitor complex was incubated with myotubes for 3 hours and the rDM64 purified by affinity chromatography showed inhibitory properties, inducing a 92% reduction of the cytotoxic effect of myotoxin II when tested at a 1:1 molar ratio (myotoxin:rDM64)(Fig 6). When a 2:1 molar ratio was used, the cytotoxicity was inhibited by 65%, whereas a 15% inhibition was observed when a 4-fold molar excess of the toxin was tested. When the inhibitory effects of equivalent concentrations of native and recombinant DM64 were compared, a slight but significant difference was observed at a single molar ratio (*i*.*e*., 2 myotoxin:1 DM64). These results indicate that both DM64 and rDM64 show similar anticytotoxic effects.

## Discussion

Molecular biology techniques enable the production of recombinant proteins in large amounts; these proteins can then be used in therapeutics and scientific research. Currently, *E*. *coli* is the most widely used expression system, although many eukaryotic proteins are not efficiently expressed in this organism. Protein misfolding and the absence of post-translational modifications are important limitations in the use of *E*. *coli*. *Pichia pastoris* could be an eukaryotic organism alternative for the expression of recombinant proteins from mammals. This methylotrophic yeast presents advantages such as the presence of a strong promoter region regulated by methanol (*AOX1*), the possibility of allowing disulfide bond formation, the ability to secrete recombinant proteins, and the capacity to perform most post-translational modifications [48–50].

The vector pPICZαA was used for rDM64 expression (Figs 1 and 2). It is regarded as a good vector because it contains the *Sh ble* gene from *Streptoalloteichus hindustanus*, which confers resistance to Zeocin, allowing the best *P*. *pastoris* clone to be selected and screened for the inhibitor expression. The recombinant inhibitor was secreted due to the presence of the N-terminal α-factor signal peptide of *S*. *cerevisiae*. The vector also encodes a C-terminal *c-myc* epitope and a polyhistidine (6xHis) tag, enabling the easy detection and purification of recombinant proteins, respectively. However, this extra 21-amino-acid sequence may modify the structure of the recombinant protein, compromising its biological activity. Santos-Filho and co-workers [51] have previously reported using the pPICZαA vector to produce recombinant BaltMIP, a myotoxin inhibitor from *B*. *alternatus* serum, adding the *c-myc* epitope and the His-tag at the C-terminus of rBaltMIP. However, the inhibitory effect of the rBaltMIP was lower than that of the native BaltMIP. For this reason, we decided to maintain the natural structure of the rDM64, and such a C-terminal peptide was not added. Despite lacking these features, our results show that rDM64 was effectively purified using affinity (column conjugated with myotoxin II) and ion exchange chromatographies (Fig 3). For the unequivocal identification of the recombinant protein, we have used mass spectrometric data instead of just epitope recognition (S1 Table).

Fermenting *Pichia pastoris* generally starts in a shake-flask system before the culture is transferred to a larger volume fermenter. The shake-flask system provides suboptimal conditions due to the lack of data recording and regulatory control systems. Nonetheless, the production of rDM64 in shake flasks was advantageous because it is a low-cost and less complex method. However, proteolytic degradation is a recurrent problem when working in shake-flask cultures. In these experiments, methanol is the carbon source and induces the *AOX1* promoter; therefore, recombinant protein induction also creates conditions that trigger an excess of protease production [48,52,53]. Methanol can also induce cell lysis by oxidative stress and heat-shock responses, eliciting a proteolytic response when the cells are growing exponentially, which results in high cell-density fermentation. In this regard, oxidative stress may also be responsible for recombinant protein degradation because of the increased amounts of reactive oxygen species that are produced during methanol induction. Our mass spectrometry analysis of protein expression in the medium identified proteases and intracellular proteins of yeast in addition to rDM64. This analysis (S1 Table) suggests that yeast lysis occurred during expression in shake flasks. The growth of *P*. *pastoris* in shake flasks created stress conditions, such as starvation, heat, pH changes, and/or toxic chemicals. Although expression in shake flasks may have also generated proteolytic products and/or glycoforms in minor quantities, it apparently did not affect rDM64 activity. The biological activity of the partially purified rDM64 fraction was similar to that of native DM64 (Fig 6).

During the expression of rDM64, we added Pefabloc, a potent and irreversible serine proteinase inhibitor, to the expression medium (Fig 1B). It has lower toxicity, improved solubility in water and higher stability in aqueous solutions than other inhibitors (*e*.*g*., PMSF and DFP). This serine protease inhibitor was used to protect against the proteolytic degradation induced by yeast serine proteases. This molecule may additionally inhibit the production of reactive oxygen species that are generated by NADPH oxidase [54–56]. Therefore, Pefabloc was also used to decrease ROS generation in yeast cultures in shake flasks, thus reducing rDM64 degradation. This report is the first to use a synthetic serine protease inhibitor during recombinant protein expression in a *Pichia pastoris* culture. However, as shown in Fig 2B, the use of Pefabloc during fermentation did not completely inhibit the proteolytic degradation of rDM64. Although its recommended working concentration ranges from 0.1 to 1 mM, a maximum concentration of 0.25 mM should be used when working with cell cultures. The concentration used in the present study (0.2 mM) may therefore represent a suboptimal inhibitor concentration considering a) the increased production of *P*. *pastoris* extracellular/cell-bound proteases that may be elicited by methanol induction and b) the release of intracellular proteolytic enzymes following cell lysis induced by oxidative stress. Nevertheless, Pefabloc favored both the growth of the cell culture and heterologous protein production, indicating that this strategy could be used for low-scale recombinant expression of other proteins.

Comparing the molecular masses of native and recombinant DM64 showed that rDM64 had a slightly smaller mass (Fig 4). Previously, our group reported that the N-glycan moiety of native DM64 is of the complex type, being composed of N-acetylglucosamine, mannose, galactose, and sialic acid [57]. However, the glycosylation of foreign proteins by *P*. *pastoris* includes only mannose residues. The N-deglycosylation assay showed that the molecular masses of the recombinant and the native protein were similar (Fig 4); hence the smaller molecular mass of the glycosylated recombinant protein is likely to be due to differences in the glycan moieties. It is important to observe that glycoproteins expressed in *Pichia pastoris* are less frequently hyperglycosylated than those produced in *S*. *cerevisiae*, although excessive glycosylation in *P*. *pastoris* has also been reported [48]. Glycosylation in the lumen of the endoplasmic reticulum after protein translation is similar in both yeast and mammalian cells, but in yeast Golgi, mannose residues are added and oligomannose units can be α-1,6 linked to the α-1,3 mannose in the Manα-1,3-Manβ-1,4-GlcNAc_2_ inner core. In this way, *P*. *pastoris* glycosylation results in diverse structural heterogeneity of the rDM64, which is attributed to the Golgi glycosylation enzymes [48,58,59]. In the heterologous expression in this study, the higher molecular mass bands (136 and 88 kDa) were also identified by mass spectrometry as rDM64 (Fig 2A). Periodic acid-Schiff (Fig 3D) results suggest that these bands may represent glycoforms that are produced by *P*. *pastoris*, although the deglycosylation assay does not seem to be conclusive (Fig 4).

Glycosylation is critical in several biological properties, including structural stability, biophysical characteristics, and resistance to proteolytic attack [60,61]. N-glycosylated proteins are abundant in eukaryotic cells, and N-linked glycans all contain a common trimannosyl-chitobiose core with one or more antennae attached to each of the two outer mannose residues [61,62]. Leon and co-workers [57] have previously shown that after sialic acid and galactose removal, DM64 was still able to interact with myotoxin II from *B*. *asper*. The same behavior was observed for DM43, an antihemorrhagic protein homologous to DM64 (71% sequence identity) that targets snake venom metalloproteases and does not inhibit myotoxins [63,64]. Interestingly, PNGase F-treated DM43 was half as effective as native DM43 in inhibiting the hydrolysis of azocasein by jararhagin [57]. On the other hand, our present results showed that the complex-type N-glycans of native DM64 can be fully replaced by a high-mannose N-glycan structure without impairing the inhibitory activity of rDM64 toward myotoxin II (Fig 6). Although other reports in the literature have shown that partial deglycosylation does not impair the structural stability of native proteins [65–67], the presence of carbohydrate moieties improves the solubility of proteins and may also be important during *in vivo* folding of nascent glycoproteins [61,68].

In summary, local myonecrosis caused by *Bothrops* species is an important public health problem, as it may cause permanent disability of the victims in addition to generating high medical costs due to increased hospitalization times [4,26,69]. This effect is mainly induced by myotoxic phospholipases A_2_ and, indirectly, by hemorrhagic metalloproteases. Therefore, the local administration of effective myotoxin inhibitors that are based on the structure of rDM64 may represent a valid alternative to reduce tissue damage at the bite site. The present study demonstrated the successful expression of rDM64 in *P*. *pastoris* cultures in small volumes. The maintenance of the native-like structure of the inhibitor was fundamental to preserving its anticytolytic effect, suggesting that rDM64 may also inhibit the *in vivo* myotoxic effect of myotoxin II. The production of a biologically active myotoxin inhibitor by yeast cells can contribute to the development of a therapeutic alternative for the treatment of envenomation by bothropic snakes. The production of rDM64 can also be exploited by studies aiming to map the structure-function relationship of toxin inhibitors and their molecular targets. This result is very important since, while many works have reported the primary structure of inhibitors isolated from snake and mammalian sera, none of these works have shown the three-dimensional structures of their inhibitors [38,63,64,70].

Our group has made multiple unsuccessful attempts to crystallize inhibitors from animal serum. Although the inhibitor DM43 has been crystallized, only low-quality diffraction patterns have been obtained. The heterogeneity of complex-type glycans tends to impair crystallization, and whether the high-mannose glycan structures inserted in recombinant proteins result in more homogeneous structures must yet be tested. The expression of DM64 in *P*. *pastoris* could also assist the development of alternative low-resolution structural analysis techniques, such as cross-linking-mass spectrometry (XL-MS), hydrogen-deuterium exchange-mass spectrometry (HDX-MS), and small-angle X-ray scattering (SAXS), using both full-length heterologous inhibitors and/or selected structural domains. Glycosylation is an important feature that maintains the structure of the inhibitor and its soluble state. Previous attempts to express toxin inhibitors using an *Escherichia coli* system were not successful, probably due to the absence of post-translational modifications. For future development, new research on rDM64 will be undertaken to increase the production scale for structural and *in vivo* assays.

## Supporting information

S1 Table MS/MS identification of *P. pastoris* fermentation products that are indicated in red in Fig 2A. (XLSX)
